# Carbon ion radiotherapy boosts anti-tumour immune responses by inhibiting myeloid-derived suppressor cells in melanoma-bearing mice

**DOI:** 10.1038/s41420-021-00731-6

**Published:** 2021-11-03

**Authors:** Heng Zhou, Pengfei Yang, Haining Li, Liying Zhang, Jin Li, Tianyi Zhang, Chengyan Sheng, Jufang Wang

**Affiliations:** 1 Key Laboratory of Space Radiobiology of Gansu Province & Key Laboratory of Heavy Ion Radiation Biology and Medicine, Institute of Modern Physics, Chinese Academy of Sciences, Lanzhou, China; 2 University of Chinese Academy of Sciences, Beijing, China; 3 Gansu Provincial Cancer Hospital, Gansu Provincial Academic Institute for Medical Sciences, Lanzhou, China; 4 Gansu University of Chinese Medicine, Lanzhou, China; 5 School of Nuclear Science and Technology, Lanzhou University, Lanzhou, China

**Keywords:** Tumour immunology, Tumour immunology

## Abstract

Numerous studies have shown that carbon ion radiotherapy (CIRT) induces anti-cancer immune responses in melanoma patients, yet the mechanism remains elusive. The abundance of myeloid-derived suppressor cells (MDSC) in the tumour microenvironment is associated with therapeutic efficacy and disease outcome. This study analysed the changes in the immune contexture in response to the carbon ion treatment. The murine melanoma B16, MelanA, and S91 tumour models were established in syngeneic immunocompetent mice. Then, the tumours were irradiated with carbon ion beams, and flow cytometry was utilised to observe the immune contexture changes in the bone marrow, peripheral blood, spleen, and tumours. The immune infiltrates in the tumour tissues were further assessed using haematoxylin/eosin staining and immunohistochemistry. The immunoblot detected the expression of proteins associated with the JAK/STAT signalling pathway. The secretion of immune-related cytokines was examined using ELISA. Compared to conventional radiotherapy, particle beams have distinct advantages in cancer therapy. Here, the use of carbon ion beams (5 GyE) for melanoma-bearing mice was found to reduce the population of MDSC in the bone marrow, peripheral blood, and spleen of the animals via a JAK2/STAT3-dependent mechanism. The percentage of CD3^+^, CD4^+^, CD8^+^ T cells, macrophages, and natural killer cells increased after radiation, resulting in reduced tumour growth and prolonged overall survival in the three different mouse models of melanoma. This study, therefore, substantiated that CIRT boosts anti-tumour immune responses via the inhibition of MDSC.

## Introduction

Tumours often escape immunosurveillance by attracting the immunosuppressive cells into the tumour microenvironment (TME) that counteract the anti-tumour immune responses [1]. Among the immunosuppressive population, myeloid-derived suppressor cells (MDSC) and regulatory T cells (Treg) are most potent, significantly affecting immune escape and disease progression [2, 3]. In humans, MDSC were initially described as immunosuppressive CD34^+^ hematopoietic progenitor cells [4] and later were shown to inhibit the activation of T effector cells and impair immunosurveillance [5]. MDSC inhibit T cell functions through well-defined mechanisms including the upregulation of arginase (ARG1) and inducible nitric oxide synthase (iNOS) [6, 7], the production of immunosuppressive cytokines, such as transforming growth factor-beta (TGF-β) and interleukin-6 (IL-6) [8, 9], as well as the sequestration of cysteine [10], and the reduced expression of L-selectin by the T cells [11]. Collectively, MDSC downregulate the expression of the anti-tumour immune molecules, promoting the evasion immunosurveillance and enhancing the proliferation and differentiation of malignant cells. In animal models and clinical studies, reducing the number of MDSC significantly improved anti-cancer immune responses in tumour-bearing mice and therapeutic outcome in cancer patients [6, 12, 13].

The Janus kinase and signal transcriptional activator (JAK2/STAT3) pathway regulates the proliferation and differentiation of MDSC [14] and also accounts for the synthesis of ARG1 and iNOS, which is the key mechanism for MDSC to inhibit T cell functions [15, 16]. The activity of MDSC can be limited by targeting its immunomodulatory function through the selective inhibition of the JAK2/STAT3 pathway. Thus, the inhibition of JAK2/STAT3 by galiellalactone led to a remarkable reduction in the activity of the cytokine GM-CSF and reduced the generation of MDSC in prostate cancer [17].

Systemic treatment with anti-cancer agents is limited by adverse toxicity and severe side effects [18]. Radiotherapy can be locally focused, imparting anti-cancer effects without the side effects of traditional chemotherapeutic approaches. Recently, carbon ion beams have gained interest for clinical use in anti-cancer therapy [19]. Compared to the conventional photon radiation, carbon ion beams are high linear energy transfer (LET) rays with superior physical properties and increased relative biological effectiveness (RBE) [20, 21]. The vast majority of energy is deposited at the Bragg peak with little to no tail dose, which causes less damage to the healthy tissue surrounding the malignant lesion [22]. Targeted carbon ion radiation induces a high frequency of double-strand breaks leaving the DNA beyond repair and serious clustered breaks leading to the death of the irradiated tumour cell [23, 24]. Taken together, the use of carbon ion beams shows great potential in the field of radiotherapy.

In recent years, several groups have reported abscopal effects in which the radiation of primary lesions not only causes the tumours to shrink but also distant metastatic lesions to disappear [25, 26]. Some studies have shown that radiation at doses of 5–20 Gy facilitates dendritic cell homing and T cell priming, thus increasing the abundance of tumour-reactive T cells [27, 28] and other studies have reported the effects on circulating cells, including bone marrow-derived myeloid cells, which can further modulate the immune response to radiotherapy [29, 30]. Altogether these results suggest that radiotherapy can yield potent anti-tumour immune responses.

Despite the positive effects of heavy ion radiotherapy on the overall survival of treated patients [31, 32], very little has been done to investigate the anti-tumour immune response on MDSC and changes in the tumour microenvironment. Immunotherapy has markedly improved the clinical outcomes in melanoma patients, thus, we used three different melanoma-bearing mouse models to investigate the anti-tumour immune responses after in situ carbon ion radiation.

## Results

### Carbon ion radiotherapy (^12^C^6+^) decreases MDSC in vivo

**Fig. 1:**
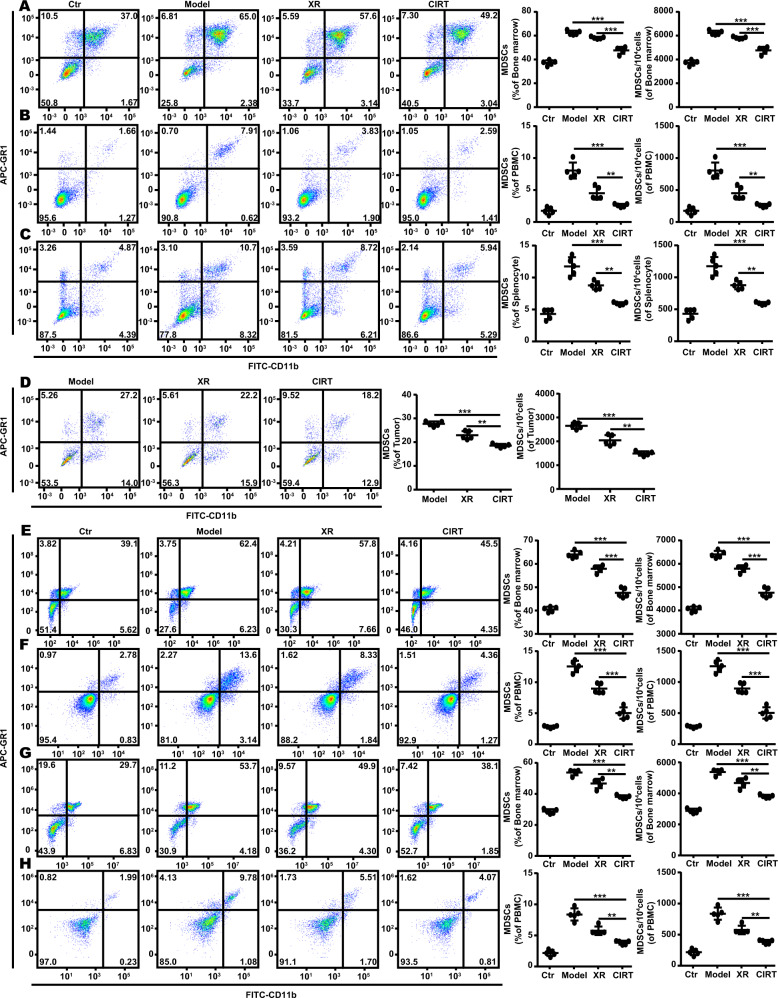
Carbon ion radiotherapy downregulates MDSC in vivo. The C57BL/6 mice (Ctr) bearing subcutaneous melanoma (model) were locally irradiated with a physical dose of 5 Gy X-ray (XR) or 5 GyE carbon ions (CIRT) on the tumour sites. On the 7^th^ day after radiation, the bone marrow, peripheral blood, spleen and tumour were harvested and stained with anti-CD11b and anti-GR1 antibodies to analyse changes in the population of CD11b^+^GR1^+^ MDSC using flow cytometry. The amount of MDSC decreased significantly in the bone marrow **A** and peripheral blood **B** and was downregulated in the spleen **C** and tumour **D** in the group that received carbon ion radiation of B16 melanoma bearing mice model. The amount of MDSC decreased significantly in the MelanA **E**, **F** and S91 **G**, **H** melanoma bearing mice model respectively in the group that received carbon ion radiation. Representative images and quantifications are shown (mean ± SD of triplicate assessments, Student’s *t*-test, **p < 0.01, ***p < 0.001).

Based on the existing knowledge of the long-term efficacy of heavy ion beams in the treatment of solid tumours, we tested whether carbon ion radiotherapy (CIRT) can decrease the abundance of the immunosuppressive cells. To achieve this, the C57BL/6 mice bearing subcutaneous B16 melanoma were locally irradiated with 5 GyE carbon ions using a heavy-ion accelerator. A 5 Gy X-ray was used as control. The percentage and numbers of MDSC in the bone marrow, peripheral blood, spleen, and tumour of the animals were assessed at 7 days after irradiation. The amount of CD11b^+^GR1^+^ MDSC in the bone marrow (Fig. 1A) and peripheral blood were much lower (Fig. 1B) and significantly decreased in the spleen (Fig. 1C) and tumour (Fig. 1D) of carbon ion-irradiated animals compared to the X-ray irradiated and non-irradiated B16 melanoma-bearing mice. Similar results were found in MelanA (Fig. 1E, F) and S91 (Fig. 1G, H) melanoma-bearing mice.

**Fig. 2:**
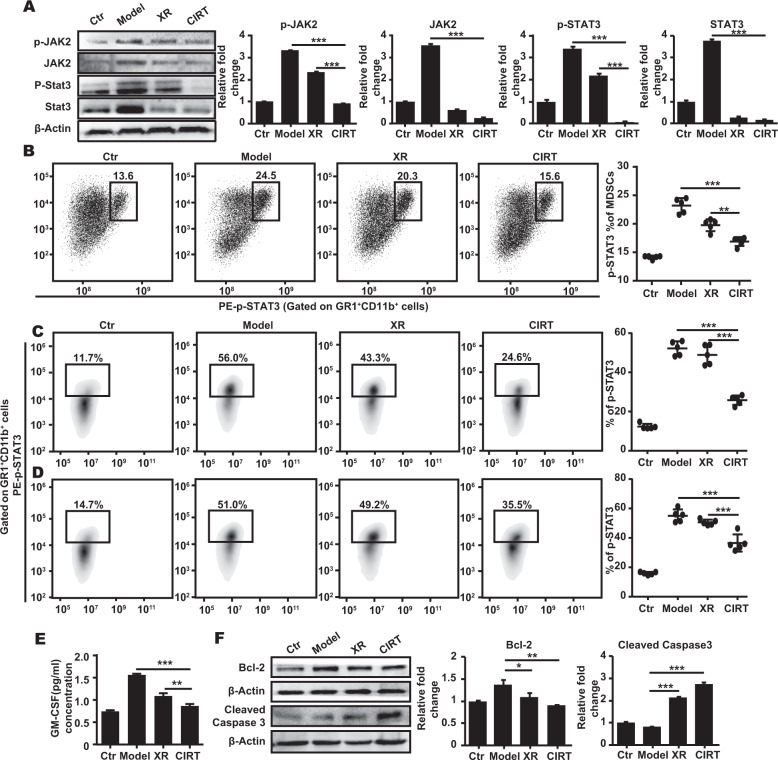
The JAK2/STAT3 signalling is involved in the downregulation of MDSC. The C57BL/6 mice (Ctr) bearing subcutaneous melanoma (model) were locally irradiated with a dose of 5 GyE carbon ions (CIRT) on the tumour sites. On the 7^th^ day after irradiation, the bone marrow cells were collected and the MDSC were sorted by flow cytometry. Following the phosphorylation of JAK2 and STAT3 was detected using phosphoneoepitope-specific antibodies using immunoblot or flow cytometry. Both p-JAK2 and p-STAT3 **A**, **B** were significantly decreased in the irradiated B16 melanoma-bearing mice. In the carbon ion radiation group, p-STAT3 decreased significantly both in MelanA **C** and S91 **D** melanoma-bearing mice. The GM-CSF was significantly decreased in the irradiated animals **E**, and the expression of the antiapoptotic protein, Bcl-2 was decreased whereas the abundance of cleaved caspase 3 was increased **F**, at the same time. Representative images and quantifications are shown (mean ± SD of triplicate assessments, Student’s *t*-test, ***p < 0.001).

Since the JAK2/STAT3 signalling pathway is known to regulate the proliferation of MDSC, we tested whether this pathway is affected by CIRT. Indeed, MDSC isolated from the bone marrow of carbon ion-irradiated animals depicted a remarkable decrease in the phosphorylation of JAK2 and STAT3 (Fig. 2A) compared to untreated model animals tested using immunoblot and additionally validated using flow cytometry (for STAT3) (Fig. 2B). Similar results were found in MelanA (Fig. 2C) and S91 (Fig. 2D) melanoma-bearing mice. We then detected the abundance of the activation factor of the JAK/STAT signalling pathway, GM-CSF and found that the secretion of GM-CSF significantly was decreased in the serum of the carbon ion-irradiated mice (Fig. 2E). Next, we looked for downstream effectors of apoptosis and found that in MDSC isolated from carbon ion-irradiated animals the expression level of the antiapoptotic protein Bcl-2 was slightly decreased and the abundance of the active form of caspase 3 was increased compared to the untreated controls (Fig. 2F). Altogether this indicates that focal carbon ion radiation decreases the abundance of MDSC through inhibition of JAK2/STAT3 signalling, resulting in apoptotic cellular demise.

### Immune response triggered by CIRT

**Fig. 3:**
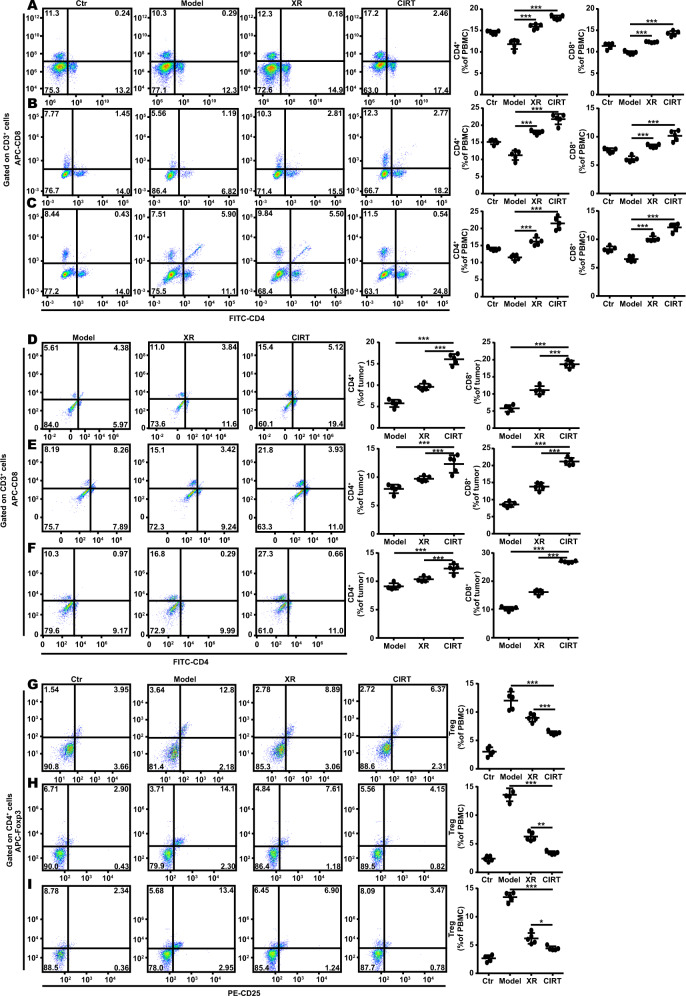
Carbon ion radiation boosts T cell proliferation in vivo. The C57BL/6 mice (Ctr) bearing subcutaneous B16, MelanA and S91 melanoma (model) were locally irradiated with the physical dose of 5 Gy X-ray (XR) or 5 GyE carbon ions (CIRT) on the tumour sites. On the 7^th^ day after radiation, the peripheral blood cells were stained with anti-CD3, anti-CD4, anti-CD8 and anti-CD4, anti-CD25, anti-FoxP3 antibodies to analyse the proliferation of T cells and Treg by flow cytometry. The abundance of CD3^+^CD4^+^ and CD3^+^CD8^+^ cells in peripheral blood and tumour tissue were increased in B16 **A**, **D**, MelanA **B**, **E** and S91 **C**, **F** mice models in irradiated group vs model animals. Treg cells were decreased in B16 **G**, MelanA **H**, and S91 **I** mice models in the irradiated group vs. model animals. Representative images and quantifications are shown (mean ± SD of triplicate assessments, Student’s *t*-test, **p < 0.01, ***p < 0.001).

Since we found that carbon ion radiation decreased the percentage of MDSC in the bone marrow, peripheral blood, and spleen of mice, we next analysed the anti-tumour T cell responses. The peripheral blood of B16/MelanA/S91 melanoma-bearing mice was extracted 7 days after radiation to analyse the T cell composition by flow cytometry. The results showed that the population of CD4^+^ and CD8^+^ in the peripheral blood of carbon ion radiation-exposed B16 (Fig. 3A), MelanA (Fig. 3B) and S91 (Fig. 3C) melanoma-bearing mice were significantly higher than that of the X-ray irradiated and untreated melanoma-bearing animals, the same results were also observed in B16 (Fig. 3D), MelanA (Fig. 3E) and S91 (Fig. 3F) tumours, while the abundance of Treg was decreased remarkably after carbon ion exposure in peripheral blood in B16 (Fig. 3G), MelanA (Fig. 3H) and S91 (Fig. 3I) melanoma-bearing mice. There was not much difference in the natural killer (NK) cells (Fig. S1A, B), whereas F4/80^+^ macrophages were increased in the peripheral blood and spleen (Fig. S1C, D) of the B16 melanoma-bearing mice.

**Fig. 4:**
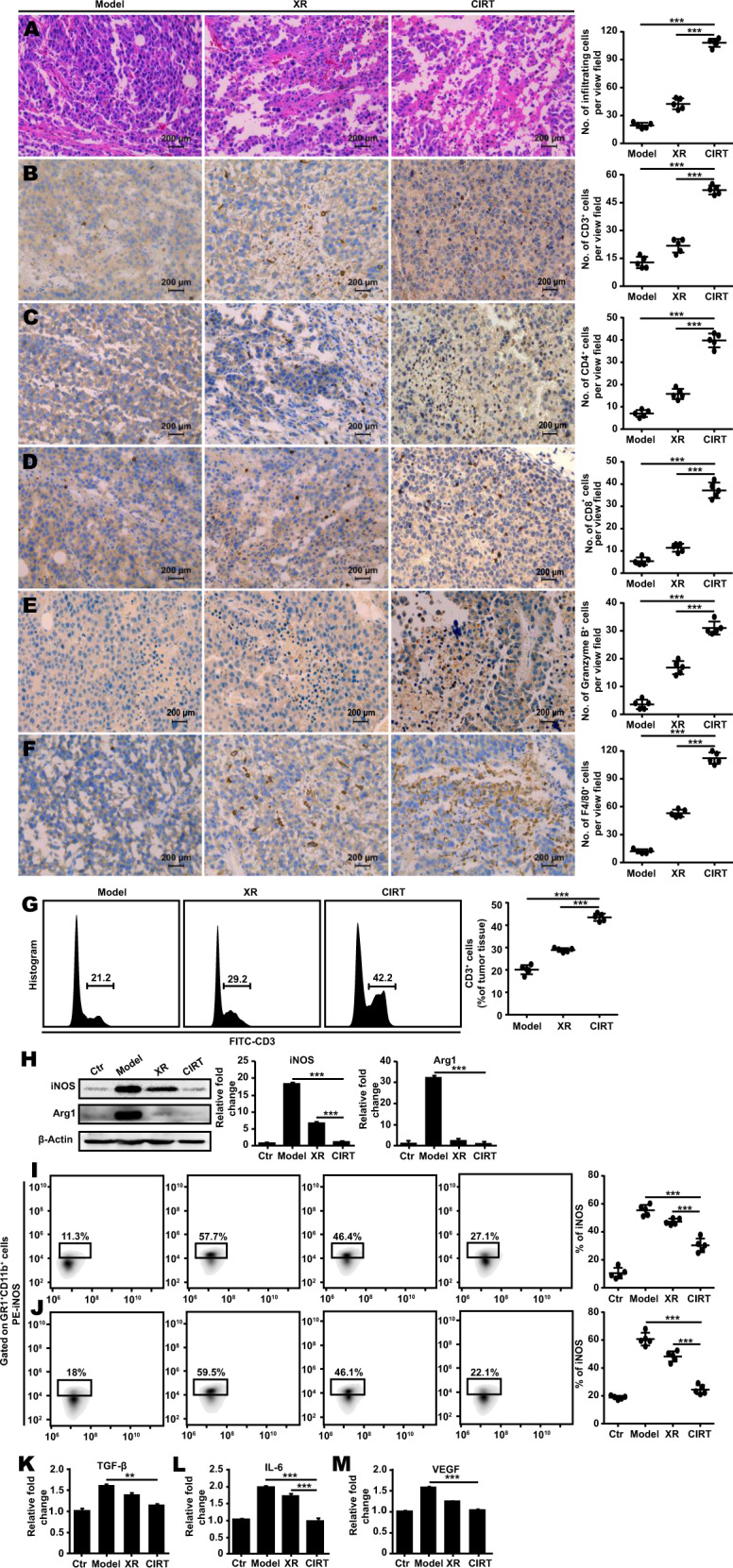
Carbon ion radiotherapy enhances the anti-tumour immune responses in vivo. The C57BL/6 mice (Ctr) bearing subcutaneous B16 melanoma (model) were locally irradiated with the physical dose of 5 Gy X-ray (XR) or 5 GyE carbon ions (CIRT) on the tumour sites. On the 7^th^ day after radiation, the tumour was excised, and the tissue was embedded with paraffin and stained with haematoxylin-eosin **A** or specific antibodies to detect the composition of the tumour immune infiltrate. The abundance of CD3^+^, CD4^+^, CD8^+^, Granzyme B^+^ and F4/80^+^ was assessed carbon ions irradiated vs X-ray irradiated and non-treated models **B–F**. Also, the quantity of CD3^+^ was assessed by flow cytometry in the dissociated tumours after the radiation in the models **G**. The expression of ARG1 and iNOS as downstream effector proteins of JAK2/STAT3 signalling pathway was assessed by immunoblot **H** and the expression of iNOS was decreased significantly both in MelanA **I** and S91 **J** melanoma-bearing mice in the group that received carbon ion radiation. The serum levels of the inflammatory cytokines-TGF-β, IL-6, VEGF were measured by ELISA **K–M**. The representative images and quantifications are shown (mean ± SD of triplicate assessments, Student’s *t*-test, *p < 0.5, **p < 0.01, ***p < 0.001).

To investigate whether CIRT can trigger anti-tumour immune response, we stained the paraffin-embedded tumour sections with haematoxylin and eosin (H&E). The microscopical analysis revealed a significant increase in the number of infiltrating leukocytes in carbon-ion irradiated tumours compared to the X-ray-irradiated and untreated B16 tumours (Fig. 4A). We then detected the abundance of CD3^+^ (Fig. 4B), CD4^+^ (Fig. 4C), CD8^+^ (Fig. 4D), Granzyme B^+^ (Fig. 4E) and F4/80^+^ cells (Fig. 4F) in the tumour by immunohistochemistry and flow cytometry (Fig. 4G). The quantity of CD3^+^, CD4^+^, CD8^+^, Granzyme B^+^ and F4/80^+^ cells were dramatically increased at day seven after carbon ion radiation compared to the X-ray irradiated and untreated B16 tumours. The significant increase in the CD8^+^, Granzyme B^+^ cells were additionally observed in MelanA and S91 tumours (Fig. S2A–D). Altogether, this indicates the onset of a specific anti-tumour immune response.

Since we found that the JAK2/STAT3 signalling pathway is implicated in the decrease of MDSC after CIRT we investigated if ARG1 and iNOS, the key downstream molecules of JAK2/STAT3, influence the proliferation of T cells upon radiation. Therefore, we detected the protein expression level by western blot and found that ARG1 and iNOS (Fig. 4H) were both significantly decreased in the carbon ion irradiated group. Besides, we also found iNOS was remarkably decreased in MelanA and S91 melanoma-bearing mice which had received carbon ion radiation (Fig. 4I, G). Moreover, using enzyme-linked immunosorbent assay (ELISA) we found that the immunosuppression-associated cytokines TGF-β (Fig. 4K), IL-6 (Fig. 4L) and VEGF (Fig. 4M) remarkably decreased in the serum of the irradiated mice. These results imply that CIRT can boost anti-tumour immune responses in mice.

### CIRT increased overall survival of tumour-bearing mice

**Fig. 5:**
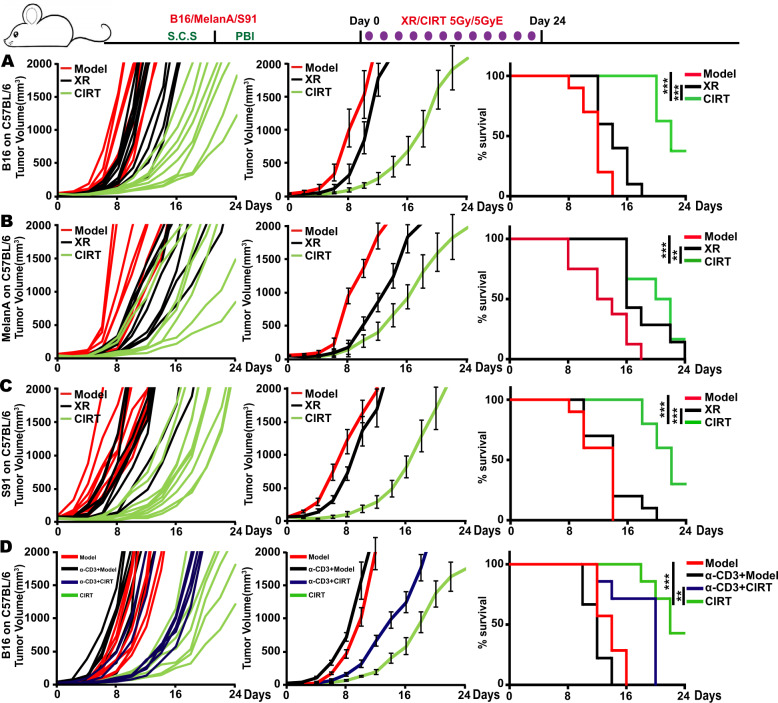
Carbon ion radiotherapy decreased tumour growth and increased overall survival. The C57BL/6 mice bearing subcutaneous B16, MelanA and S91 melanoma (model) were locally irradiated with a physical dose of 5 Gy X-ray (XR) or 5 GyE carbon ions (CIRT) on the tumour site. The tumour volume of the mice was measured every 2^nd^ day from the day of radiation until the tumour volume reached 2000 mm^3^. The carbon ion radiotherapy prolonged the survival time of tumour-bearing mice and slowed down the growth rate of tumours **A–C**. CD3 T cell-depleted littermates bearing subcutaneous B16 melanoma were locally irradiated with physical dose of 5 Gy X-ray (XR) or 5 GyE carbon ions (CIRT) on the tumour site. The tumour growth in the CD3-depleted mice was faster than in an immunocompetent group, the carbon ion radiation decreased the overall survival though promoting immune response **D**. Representative images and quantifications are shown (mean ± SD of triplicate assessments, Student’s *t*-test, ***p < 0.001).

To explore the effect of CIRT on tumour growth and overall survival of melanoma-bearing mice, we transplanted B16, MelanA and S91 three different melanoma cells into immunocompetent syngeneic C57BL/6 hosts. When the tumours became palpable (20 mm^3^), CIRT and X-ray irradiation with 5 GyE/5 Gy was applied on tumour site. Following irradiation, we compared the tumour size of the carbon ion-irradiated versus X-ray-irradiated and non-irradiated mice and found that the tumour growth was significantly reduced, and the overall survival was remarkably prolonged after CIRT compared to the X-ray-irradiated and untreated animals (Fig. 5A–C). The CD3-depleting antibodies were intraperitoneally injected into the B16 melanoma-bearing animals, to establish an “immunodeficient” tumour-bearing mouse model. After carbon ion irradiation, we found that the tumour growth in the CD3-depleted mice was faster than in the immunocompetent group, resulting in a decreased overall survival (Fig. 5D). Taken together, CIRT induces changes in the immune contexture in melanoma-bearing mice from an immunosuppressive to an immunogenic type, decreasing tumour growth and positively affecting the overall survival.

### Discussion and Conclusion

Our study suggests that CIRT can cause a decrease in MDSC, and the onset of anti-tumour immune responses, resulting in a decrease in the tumour growth and an increase in the overall survival of the melanoma-bearing mice. Chen et al. reported an increase in the population of MDSC in the peripheral blood of the patients with head and neck tumour after 50 Gy high-dose multi-fractionated stereotactic body radiation therapy (SBRT). The number of MDSC was reduced only when the radiation was combined with the receptor tyrosine kinase inhibitor sunitinib [12]. However, another study by Yang-Xin Fu and colleagues showed that in CT26 and MC38 colon cancer models, high-dose of radiation (12 Gy) reduced the number of MDSC [33]. Since the RBE of carbon ion beams is higher than those of photons, we achieved similar effects on MDSC with only a single fraction of 5 GyE carbon ions. Numerous studies have reported that certain chemotherapeutic agents were able to reduce the population of MDSC by inhibiting the phosphorylation of JAK2 and STAT3 [34, 35]. Here, we showed that CIRT induced a decrease in the phosphorylation levels of JAK2 and STAT3 while the expression of anti-apoptotic Bcl-2 was reduced and the caspase activity was increased which might be explained why the percentage and number of MDSC decreased. Moreover, we observed a significant decrease in GM-CSF, IL-6, TGF-β and VEGF in the serum of post-radiation mice, which resulted in a decrease of JAK2 and STAT3 phosphorylation, ultimately leading to a decrease in the number of MDSC. This is consistent with studies reporting that the suppression of GM-CSF secretion from tumour cells induced a STAT3-dependent inhibition of liver-MDSC generation [36]. We further showed that CIRT was able to increase the abundance of CD4^+^ and CD8^+^ T lymphocytes in melanoma-bearing mice, increased the population of macrophages and natural killer cells and reduced the percentage of Treg. Similarly, it has been found in other studies that irradiation can increase the abundance of immune cells in the periphery [28, 33, 37]. Fan Yang and Susanne M Steggerd illustrated that the inhibition of iNOS and Arg1 can block myeloid cell-mediated immune suppression respectively [38, 39], these findings are also consistent with our conclusions. Therefore, we believe that CIRT is sufficient to boost anti-cancer immune responses while causing less injury to the surrounding tissues, thus posing less adverse side-effects. Altogether CIRT offers an immunostimulatory anti-cancer regimen that in general is well tolerated by tumour patients. In our study, we found that CIRT can decrease tumour growth while increasing overall survival, yet it failed to eliminate cancer. Nevertheless, some studies showed that in animal models with spontaneous breast tumours (TUBO tumours) or subcutaneously implanted colon cancer cells (MC38), combining anti-PD-L1 with radiation (12 Gy) therapy can effectively inhibit the growth of tumour cells [33]. Moreover, local upregulation of the PD-L1/PD-1 axis following radiation therapy suppresses radiation-induced immune responses, thereby facilitating tumour relapse [40]. Therefore, we hypothesise that the combination treatment of carbon ion radiation with PD-1/PD-L1 antibody may further improve the positive immune response in mice and might achieve tumour clearance and long-term disease control.

**Fig. 6:**
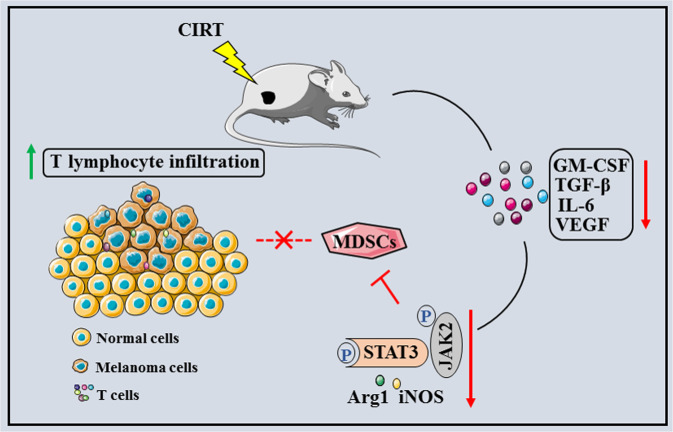
Schematic of the mechanism of CIRT boosts anti-tumour immune responses in melanoma-bearing mice. Carbon ion radiotherapy enhances anti-tumour immune responses by inhibiting myeloid-derived suppressor cells through a JAK2/STAT3-dependent signalling pathway.

In summary (Fig. 6), we conclude that CIRT can reduce the population of MDSC through a JAK2/STAT3-dependent signalling pathway, boost anti-tumour immune responses, and reduce tumour growth, therefore, increasing the overall survival of the melanoma-bearing mice.

## Materials and methods

### Radiotherapy

Carbon ion (^12^C^6+^) beam radiation was performed at the treatment terminal of the Heavy Ion Research Facility in Lanzhou (energy: 80 MeV/u, peak LET: 50 KeV/μm, SOBP). The X-ray was generated by an X-Rad 225 generator (Precision) (energy: 225 KV/13.3 mA). The RBE value of carbon ions was 1.7 times higher for X rays at a LET of around 50 KeV/μm, that means 5 GyE is a dose of 2.94 Gy. The melanoma-bearing mice were placed on the platform after anaesthesia, and only the tumour site was exposed to radiation.

### The cell lines

The B16 cell line (C57BL/6 mouse melanoma) and S91 cell line (C57BL/6 mouse melanoma) was obtained from ATCC. Melan A cell line (mouse melanoma) was obtained from the NCACC. The cell lines were cultured in DMEM with 10% FBS. All the cell lines were verified as being free of microbial contamination.

### Western blot

Half a million CD11b^+^ GR1^+^ cells were collected from the bone marrow of several mice (unirradiated leg) in the same group by flow cytometry, resuspended in the lysis buffer containing 150 mM sodium chloride, 1.0 % NP-40, 0.5% sodium deoxycholate, 0.1% SDS and protease inhibitor cocktails (Roche, Basel, Switzerland) and incubated on ice for 30 min. The cell lysate was centrifuged at 12000 g for 10 min at 4 °C to remove the insoluble material. Then the lysate was mixed with 4× NuPAGE® LDS Sample Buffer and 10×Sample Reducing Agent and proteins were denatured at 100 °C for 10 min. The NuPAGE® Novex® 4–12% Bis-Tris Protein Gels (Thermo Fisher Scientific, Waltham, MA, US) were used for protein electrophoresis under a 100 V constant voltage mode. The separated proteins were transferred from the gel to the PVDF membrane (Merck-Millipore, Darmstadt, Germany). After blocking with 5% BSA in 1× TBS containing 0.1% Tween^®^-20 (1× TBST) for 1 h at 24 °C, the membranes were probed with the corresponding primary antibodies at 4 °C overnight: anti-STAT3 antibody (ab119352, Abcam), anti-p-STAT3 antibody (ab76315, Abcam), anti-JAK2 antibody (ab205223, Abcam), anti-p-JAK2 antibody (ab32101, Abcam), anti-Bcl-2 antibody (#3869, CST), anti-Cleaved-Caspase3 antibody (#9664, CST), anti-Cyclin D1 antibody (#2978, CST), anti-iNOS antibody (MAB9502, R&D Systems), anti-ARG1 antibody (AF5868, R&D Systems). The membranes were then washed and incubated with the HRP-conjugated secondary antibodies (SouthernBiotech) at 24 °C for 2 h. The peroxidase activity was detected with the ECL Western Blotting Detection Reagent (GE Healthcare) and the images were acquired using an ImageQuant LAS 4000 (GE healthcare).

### Flow cytometry

The mice were sacrificed by cervical dislocation and the erythrocytes in the bone marrow, peripheral blood (collected in a heparin-lithium blood anticoagulant tube), splenocytes and the tumour tissue were lysed with red blood cell lysis buffer (#07800, Stem Cell Technologies). The peripheral blood mononuclear cell (PBMC) were enriched by density gradient centrifugation using the Ficoll-Paque Plus (#45-001-749, GE Healthcare). The tumour tissue cells were digested with Gentle Collagenase/Hyaluronidase (#07919, STEMCELL Technologies) and placed at 37 °C and 5% CO_2_ in a constant temperature incubator with saturated humidity for 30 min. Single-cell suspensions were prepared by gentle teasing through disposable sieves (70 μm) into cold PBS. Then cells were spun at 800 rpm for 10 min supernatant was discarded and 1 × 10^5^ cells or 1 × 10^7^ (tumour tissue cells) were resuspended in the cell staining buffer (#420201, Biolegend) containing surface antibodies and incubated in the dark at 4 °C for 30 min. The intracellular permeabilisation procedure for p-STAT, iNOS, and FoxP3 staining were implemented with the True-Nuclear™ Transcription Factor Buffer Set (#424401, Biolegend) following the manufacturer’s instructions. The antibodies FITC-CD11b(#101206), APC-GR1 (#108412), FITC-CD3 (#100204), PE-CD3 (#100206), FITC-CD4 (#100406), APC-CD8b.2 (#140410), PE-FoxP3 (#126404), AlexaFluor488-NK1.1 (#108718), APC-F4/80 (#123116) were purchased from Biolegend, PE-p-STAT3(#12-9033-42), PE-iNOS (#12-5920-82) were purchased from eBioscience^TM^ (Carlsbad, CA, US). The expression of surface and intracellular molecules was detected using a Beckman MoFloAstrios EQ flow cytometer. The gating strategies were shown in Fig. S3A, B.

### Determination of cytokines in serum

The quantification of cytokines in mice serum was performed using an enzyme-linked immunosorbent assay (ELISA): GM-CSF kit (#1217302, DAKEWE), VGEF ELISA kit (#1217342, DAKEWE), TGF-β ELISA kit (#1217102, DAKEWE) and IL-6 ELISA kit (#1210602, DAKEWE), following the manufacturer’s instructions. The absorbance was analysed using an i3 Paradigm multi-label reader (Molecular Devices).

### The experiments on the mice model

Male wild-type C57BL/6 mice at the age of 6–8 weeks were obtained from the Lanzhou Veterinary Research Institute, Chinese Academy of Agricultural Sciences and maintained in the animal facility at the Gansu University of Chinese Medicine in specific pathogen-free conditions in a temperature-controlled environment with 12 h light, 12 h dark cycles and received food and water *ad libitum*. All the animal experiments were approved by the Ethical Committee of the Gansu University of Chinese Medicine and followed EU Directive 2010/63/EU guidelines. B16, MelanA and S91 tumours were established in C57BL/6 hosts by subcutaneously inoculating 500,000 cells. When the tumours became palpable (20 mm^3^) they were locally irradiated with 5 GyE of carbon ion beams or 5 Gy X-ray. Anti-CD3 i.p. injections were repeated every 3 days to assure the complete depletion of both T cell populations during the whole experiment. Tumour-bearing mice were frequently monitored, and tumour growth was documented regularly. Following the ethical committee, advice mice were sacrificed when tumour size reached ethical end-points or signs of obvious discomfort associated with the treatment were observed.

### Immunohistochemistry

For the immunohistochemical staining of CD3 (ab5690, Abcam), CD4 (ab183685, Abcam), CD8 (ab209775, Abcam), Granzyme B (ab4059, Abcam) and F4/80 (ab111101, Abcam), 3 μm sections were cut from the paraffin blocks and placed on positively charged slides. The primary antibody was used at a dilution of 1:200. The detection kit Lab Vision™ UltraVision™ Quanto Detection System (#TL-060-QAL, Thermo Fisher Scientific) was utilised together with 3,3′-diaminobenzidine tetrahydrochloride (DAB) as the chromogen. The paraffin-embedded tumours were used for H&E staining according to standard procedures. Five fields were counted per slide in every simple. The percentage of phenotypically altered cells was evaluated using the ImageJ software (http://imagej.nih.gov/ij/).

### Statistical analysis

Unless otherwise specified, the experiments were performed in triplicates and repeated at least once. The data were analysed and the histograms were generated using the GraphPad Prism 7 software. The statistical differences were determined using a 2-way ANOVA analysis followed by the Bonferroni’s test comparing with the controlled conditions (*p < 0.05, **p < 0.01 and ***p < 0.001).

## Supplementary information

Supplementary Figures X Figure Legends

## Data Availability

All data generated and analysed during this study are included in this article. Each experiment was performed at least three times independently.
