# Supplementary material for: Carbon ion radiotherapy boosts anti-tumour immune responses by inhibiting myeloid-derived suppressor cells in melanoma-bearing mice
Source: Cell Death Discov. 2021 Nov 3;7:332. doi: 10.1038/s41420-021-00731-6 (PMC8566527; doi:10.1038/s41420-021-00731-6)
Supplement: Supplementary file 1 — Supplementary Figures X Figure Legends [file 41420_2021_731_MOESM1_ESM.pdf]

Figure S1. Carbon ion radiation affects the population of immune cells.

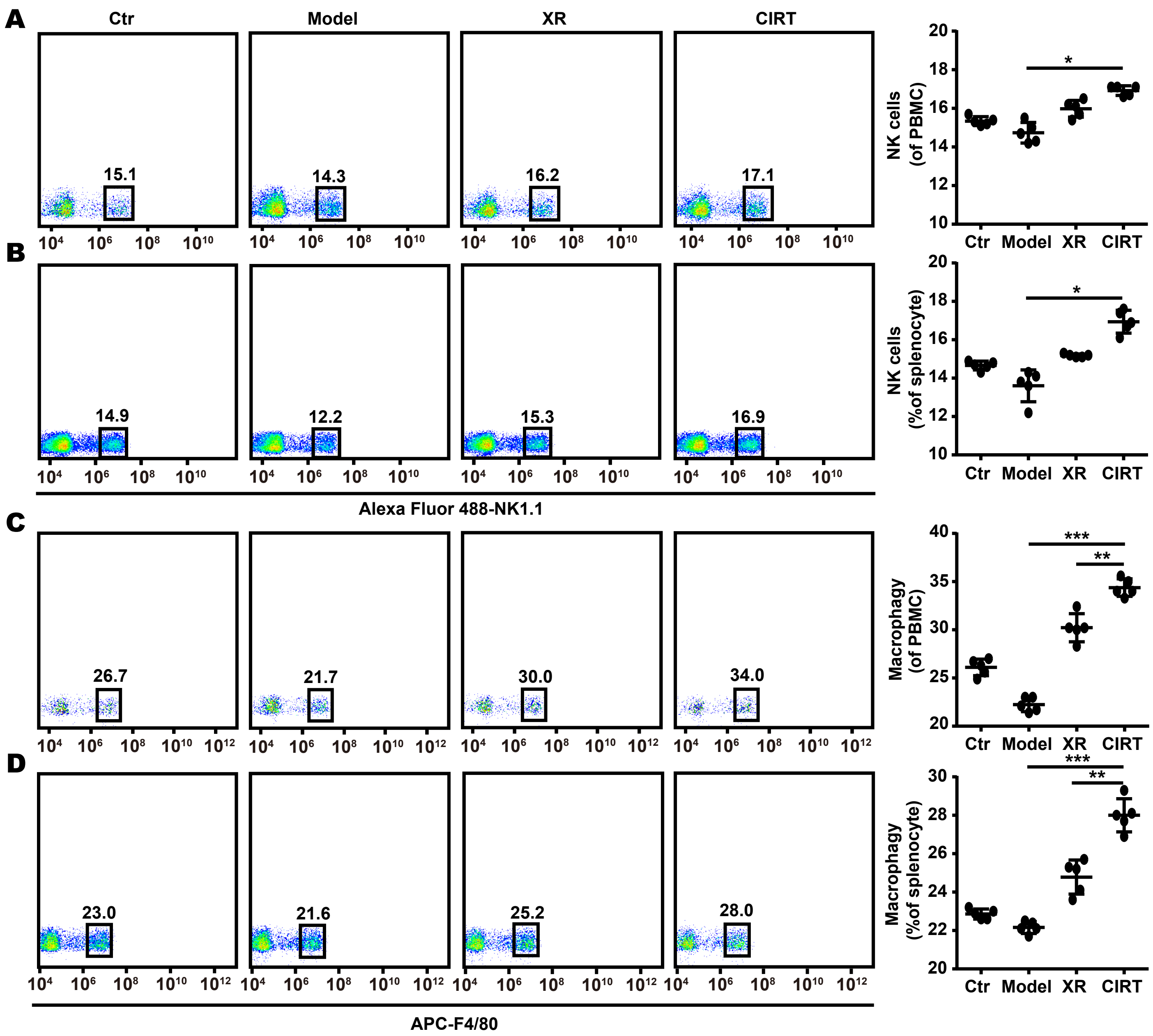

Figure S2. Carbon ion radiation enhances the infiltration and activation of CD8+ T lymphocyte in vivo.

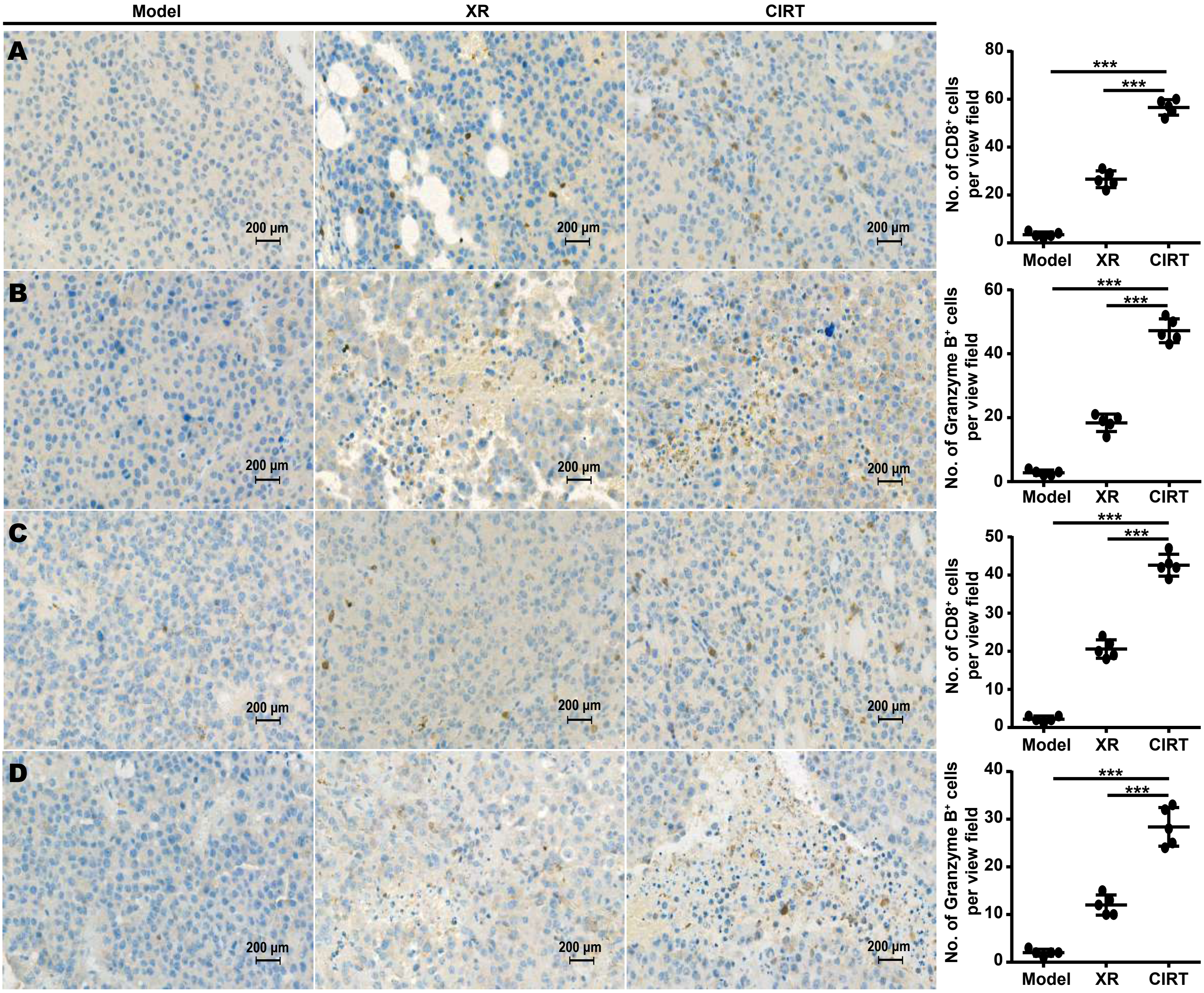

Figure S1. The gating strategies for flow cytometric data.

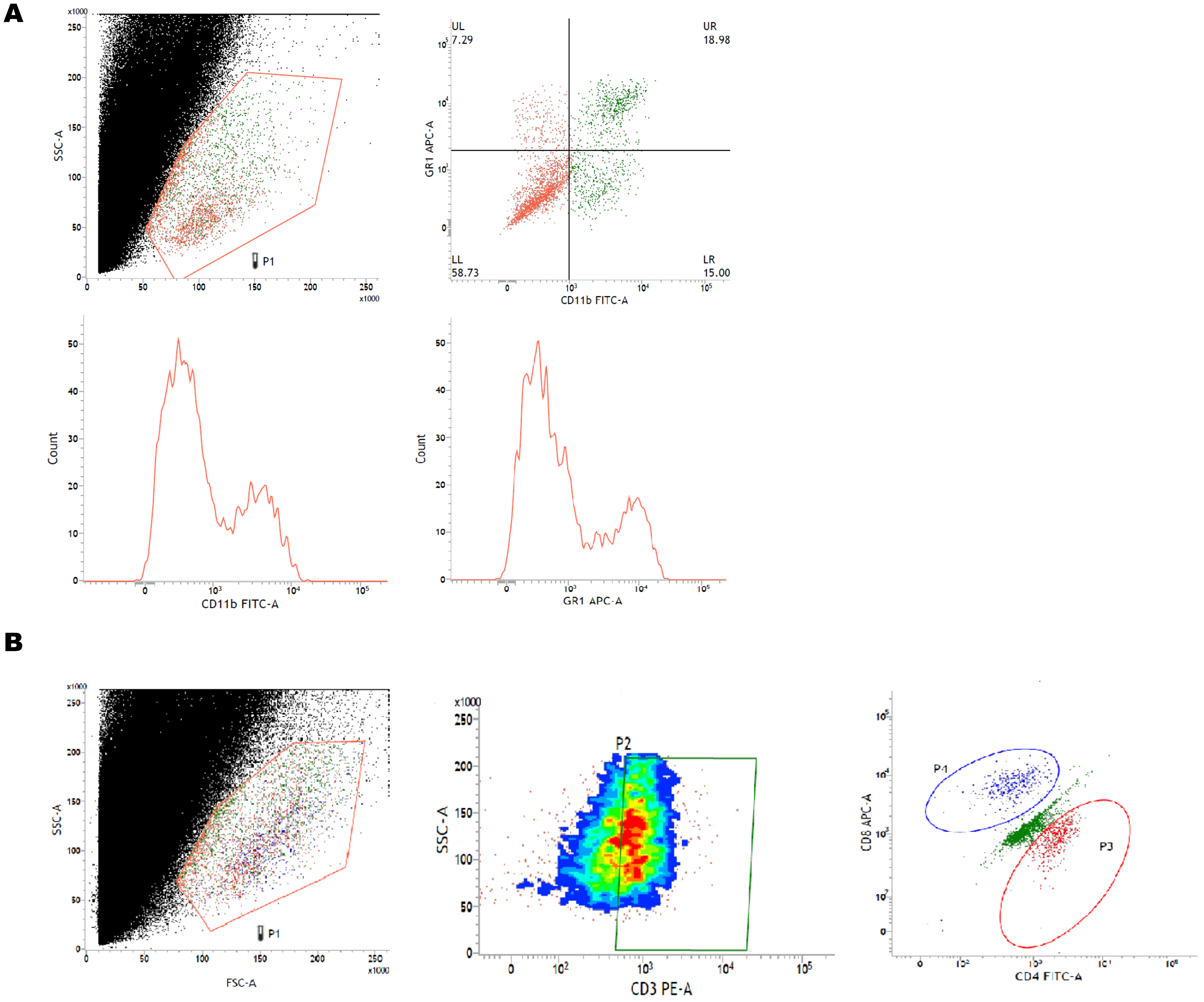

Supplementary Figures

Figure S1. Carbon ion radiation affects the population of immune cells.

The C57BL/6 mice (Ctr) bearing subcutaneous B16 melanoma (model) were locally irradiated with the physical dose of 5 Gy X-ray (XR) or 5 GyE carbon ions (CIRT) on the tumour sites. On the 7th day after radiation, the peripheral blood cells and spleen cells were stained with the corresponding antibodies to analyse changes in the NK cells and macrophage populations using flow cytometry. The abundance of NK cells (**A, B**) and macrophage was increased in the carbon ion radiation group (**C, D**). Representative images and quantifications are shown (mean ± SD of triplicate assessments, Student's *t*-test, *$p < 0.05$, ***$p < 0.001$).

Figure S2. Carbon ion radiation enhances the infiltration and activation of $CD8^+$ T lymphocyte *in vivo*.

The C57BL/6 mice bearing subcutaneous MelanA and S91 melanoma (model) were locally irradiated with the physical dose of 5 Gy X-ray (XR) or 5 GyE carbon ions (CIRT) on the tumour sites. On the 7th day after radiation, the tumour was excised, and the tissue was embedded in paraffin and stained with specific antibodies to detect the composition of the tumour immune infiltrate. The abundance of $CD8^+$ and Granzyme B^+ were significantly increased in the carbon ions-irradiated MelanA (**A, B**) and S91 (**C, D**) group vs the X-ray irradiated and non-treated models. Representative images and quantifications are shown (mean ± SD of triplicate assessments, Student's *t*-test, ***$p < 0.001$).

Figure S3. The gating strategies for flow cytometric data.

The C57BL/6 mice bearing subcutaneous B16 were locally irradiated with 5 GyE carbon ions on the tumour sites. The tumour cells were stained with anti-CD11b, anti-GR1 and anti-CD3, anti-CD4, anti-CD8 antibodies to analyse the proportion of MDSCs (**A**) and T cells (**B**) by flow cytometry.
